# Supplementation with a juice powder concentrate and exercise decrease oxidation and inflammation, and improve the microcirculation in obese women: randomised controlled trial data

**DOI:** 10.1017/S0007114513001001

**Published:** 2013-04-16

**Authors:** Manfred Lamprecht, Georg Obermayer, Kurt Steinbauer, Gerhard Cvirn, Lidija Hofmann, Gerhard Ledinski, Joachim F. Greilberger, Seth Hallstroem

**Affiliations:** 1 Institute of Physiological Chemistry, Centre for Physiological Medicine, Medical University of Graz, Harrachgasse 21/II, 8010 Graz, Austria; 2 Institute of Nutrient Research and Sport Nutrition, Petersbergenstrasse 95b, 8042Graz, Austria; 3 SportchirurgiePlus, Centre for Individual Sport Medicine and Surgery, Berthold Linderweg 15, 8047Graz, Austria; 4 FH JOANNEUM, University of Applied Sciences, Eggenberger Allee 11, 8020Graz, Austria; 5 Institute of Laboratory Sciences, Dr Greilberger GmbH, Hauptstrasse 140, 8301Laßnitzhöhe, Austria

**Keywords:** Dietary supplements, Exercise and obesity, Oxidation and inflammation, Skin microcirculation

## Abstract

Obesity and sedentary lifestyle are associated with increased oxidative stress, inflammation and vessel dysfunction. Previous research has shown that an encapsulated fruit/berry/vegetable juice powder (FBV) supplement or controlled exercise training improve the markers of redox biology, low-grade inflammation and circulation. The aim of the present study was to assess the effects of 8 weeks of supplementation with FBV or placebo, and a single bout of controlled walking on the markers of oxidation, inflammation and skin capillary microcirculation in forty-two obese pre-menopausal women (41 (sd 5) years, non-smokers and BMI 34·5 (sd 3·8) kg/m^2^) using a randomised, double-blind, placebo-controlled design. All assessments were made before and after 8 weeks of capsule supplementation, and pre- and post-30 min of controlled treadmill walking at 70 % of VO_2max_. Venous blood was collected for the determination of carbonyl proteins (CP), oxidised LDL (ox-LDL), total oxidation status (TOS) of lipids, malondialdehyde, TNF-α and IL-6. Capillary blood flow, O_2_ saturation of Hb (SO_2_Hb) and the relative concentration of Hb (rHb) were assessed at a 2 mm skin depth. Following 8 weeks of supplementation, compared with placebo, the FBV group had a significant (*P*< 0·05) reduction in CP, ox-LDL, TOS and TNF-α, and a significant increase in blood flow, SO_2_Hb and rHb. Independent of supplementation, moderate exercise significantly increased blood flow and rHb, with a trend towards increased SO_2_Hb. Compared with placebo, 8 weeks of supplementation with FBV decreased the markers of systemic oxidation and inflammation. Both FBV supplementation and a single walking bout improved the markers of the microcirculation in these obese women.

In recent decades, increasing prevalence of obesity has become a serious public health concern. Suboptimal dietary habits coupled with a sedentary lifestyle are thought to be major contributors to this situation. Overweight and obesity are associated with irregularities in redox homeostasis, imbalanced pro-inflammatory and anti-inflammatory states and microcirculatory dysfunction^(^ ^1^ ^–^ ^3^ ^)^. Reactive Oxygen and Nitrogen species and pro-inflammatory cytokines from both visceral and subcutaneous fat compartments are implicated in increased cardiometabolic disease risk^(^ ^4^ ^,^ ^5^ ^)^. Recent research has shown that obese people have structural and functional alterations in skin microcirculation, which are proportional to the increase in the degree of global and central obesity^(^ ^6^ ^)^. Hence, diets rich in antioxidants and anti-inflammatory nutrients, as well as physical exercise, are of interest to combat some of the detrimental side effects of overweight and obesity.

It has been reported that increased consumption of fruits and vegetables improves the body's antioxidant and anti-inflammatory capacities^(^ ^7^ ^–^ ^9^ ^)^. Nutraceuticals providing phytochemicals and vitamins, such as an encapsulated fruit and vegetable juice powder concentrate, have also demonstrated beneficial effects on the markers of oxidative stress, inflammation and skin microcirculation^(^ ^10^ ^–^ ^12^ ^)^.

Similar effects are also observed with exercise. Regular exercise training and single bouts of exercise improve redox biology, exert anti-inflammatory effects and are able to enhance the microcirculation in different populations, including overweight subjects^(^ ^13^ ^–^ ^16^ ^)^.

However, the influence of both exercise and nutrient supplementation on oxidation, inflammation and skin microcirculation in a target group of obese women has not been established.

Thus, the primary objective of the present study was (1) to explore the effects of a fruit, berry and vegetable juice powder concentrate (FBV) on oxidation, inflammation and skin microcirculation, compared with placebo. The secondary and tertiary goals were (2) to evaluate whether a single bout of defined walking exercise – with or without the FBV treatment – affects the capillary microcirculation from the skin surface to a 2 mm depth, and (3) to evaluate whether a defined model of walking exercise generates oxidative stress – with or without the FBV treatment – in a cohort of obese but otherwise healthy pre-menopausal women.

## Experimental methods

### Study population

A total of forty-two overweight and obese pre-menopausal women participated in the present trial. Inclusion criteria were as follows: female; age 35–50 years; regular menses; able to participate in walking exercise; non-smokers; sedentary work and lifestyle; BMI between 28 and 40 kg/m^2^; no dietary or nutritional supplement use within the 4 weeks before the first exercise test. Exclusion criteria included the following: smokers; women who failed exercise eligibility testing – as described by the Austrian and German standards in sports medicine^(^ ^17^ ^)^; chronic or excessive alcohol consumption; pregnancy and/or lactation; recent surgery or illness; diabetes; dyslipidaemia; current participation in a weight management programme; diagnosis of osteoporosis or osteopenia; current use of any medication known to significantly influence inflammation, redox biology or haemostasis. In addition to these inclusion and exclusion criteria, a standard blood chemistry panel, exercise echocardiography and maximum O_2_ uptake (VO_2max_) were determined in all women to confirm general health before study enrolment. All subjects also completed a medical history and a physical activity/well-being questionnaire.

### Ethical aspects, recruitment and randomisation

The present study was conducted according to the guidelines laid down in the Declaration of Helsinki, and all procedures involving human subjects were approved by the Ethical Review Committee of the Medical University of Graz, Austria. All subjects provided written informed consent before participating in the present investigation. The trial was registered at www.clinicaltrials.gov (identifier no. NCT01476033).

The study focused on office workers and was announced in local newspapers. A telephone screening conducted by study staff resulted in fifty-nine volunteers for further eligibility testing. Among these volunteers, forty-four women met the inclusion and exclusion criteria and were enrolled.

Subjects were randomised in blocks of six and sequentially numbered (www.randomization.com). To guarantee a balanced BMI distribution between the groups (FBV or placebo), we conducted stratification via BMI rank statistics. The randomisation code was held by a third party (Union of Sport and Exercise Scientists Austria) and provided for statistical analyses of the complete dataset.

### Study design and time schedule

This was a randomised, double-blind, placebo-controlled study. All eligibility testing was finalised 4 weeks before the baseline controlled walking bout. On that morning, a standardised breakfast (2–3 h before exercise) was provided. Then, each subject came to the laboratory to perform her 30 min exercise test at an intensity of 70 % of individual VO_2max_. After the test, the investigator dispensed the randomised capsule supply according to the subject's BMI ranking. Following 8 weeks of capsule supplementation as directed, they returned their remaining capsules and the same test procedure was repeated. All subjects were checked by a physician before each exercise test. The walking tests were scheduled between days 10 and 20 of the menstrual cycle.

### Dietary parameters

Subjects were instructed to maintain their habitual diet and lifestyle during the 8-week study and to duplicate their diet before each exercise testing/blood collection appointment, as described below. Before the first 30 min walking test, subjects completed a 7 d food record to assess nutrient intake. Subjects subsequently received copies of their 7 d diet records and were instructed to replicate the diet before the second exercise test. The standardised breakfast was served 2–3 h before both exercise tests to limit nutrient variation due to self-selection on the morning scheduled for blood collection. The standardised breakfast consisted of 250 ml low-fat yogurt, 10 g butter, 20 g jam or honey, 50 g rye-wheat bread and 500 ml of plain water, providing 1500 kJ, 13 g protein, 47 g carbohydrate and 13 g fat. Diet records were analysed for total energy, protein, carbohydrate, fat, cholesterol, fibre, water, alcohol and several vitamins, minerals and fatty acids using Opti Diet software 5.0 (GOEmbH).

### Study capsules

Women randomised to the FBV group (*n* 22) received capsules containing primarily a blended fruit, vegetable and berry juice powder concentrate derived from the following: acerola cherry, apple, bilberry, blackberry, black currant, blueberry, beetroot, broccoli, cabbage, carrot, Concord grape, cranberry, elderberry, kale, orange, peach, papaya, parsley, pineapple, raspberry, red currant, spinach and tomato (Juice Plus+^®^ Premium; NSA), as described previously^(^ ^10^ ^)^. Briefly, the FBV capsules provided 7·5 mg β-carotene, 200 mg vitamin C, 60 mg RRR-α-tocopherol, 600 μg folate and 63 kJ/d. Those subjects randomised to the placebo group (*n* 22) received identically appearing opaque white capsules containing microcrystalline cellulose. All subjects were instructed to take three capsules twice daily with meals, in agreement with the label use instructions for the retail product, for a total of six capsules per d.

### Eligibility exercise test

As part of eligibility testing, each subject performed an incremental exercise test on a treadmill ergometer (QUASARmed; HP Cosmos Sports & Medical GmbH) to check the heart and circulatory function and for the determination of VO_2max_. A standard electrocardiogram was recorded throughout all exercise tests, which were supervised by a physician. Respiratory gas exchange variables were measured throughout the incremental exercise tests using a breath-by-breath mode (Metalyzer 3B; Cortex Biophysik GmbH).

### Endurance exercise test

For the 30 min aerobic exercise tests, the walking speed was adjusted to 70 % of individual VO_2max_ on the treadmill ergometer after the standardised breakfast described previously. All tests were performed on the same treadmill, with the same standardised room temperature (20°C) and humidity (60 %). Blood pressure was measured at the beginning and every 10 min until the bout was completed.

### Blood collection and sample preparation

At each laboratory visit, two EDTA blood samples were collected from each participant, in a supine position, from a medial cubital vein: before exercise (pre) and immediately post-exercise (post). This venous blood was collected to determine the concentrations of carbonyl proteins (CP), oxidised LDL (ox-LDL), total oxidation status of lipids (TOS), malondialdehyde (MDA), TNF-α and IL-6. After centrifugation for 10 min, plasma was removed and frozen at − 70°C until analysis.

### Biochemical analyses

CP concentration was quantified using ELISA methods developed previously by Buss & Winterbourn^(^ ^18^ ^)^ and Alamdari *et al.* ^(^ ^19^ ^)^. These methods are based on the antibody recognition of carbonyl protein-bound 2,4-dinitrophenylhydrazine.

For ox-LDL determination, a commercially available immunosorbent kit (Mercodia AB) based on a direct sandwich technique was utilised.

The TOS assay determines total lipid peroxides (Immundiagnostik AG) by the detection of a coloured product from the reaction of a peroxidase with the peroxides in the sample, followed by the conversion of tetramethylbenzidine.

MDA concentration was determined according to a previously described HPLC method by Pilz *et al.* ^(^ ^20^ ^)^ after derivatisation with 2,4-dinitrophenylhydrazine.

Both TNF-α and IL-6 concentrations were analysed using commercially available ELISA kits with monoclonal antibodies (TNF-α: Immundiagnostik AG; IL-6: Invitrogen; LifeTech Austria).

### Microcirculation parameters

Measurements were conducted in a supine position after a 10 min rest before and after the walking exercise bout, on the back of the hand, between the first and second metacarpal bone. This tissue photo spectrometry technology is also called ‘oxygen to see’ (Lea Instruments). All measurements were performed by the same technician. A laser Doppler effect, as described elsewhere^(^ ^12^ ^,^ ^21^ ^)^, was used to determine microcirculatory blood flow. For the determination of O_2_ saturation of Hb (SO_2_Hb) and relative Hb concentration (rHb), white-light tissue spectrometry was utilised: SO_2_Hb is identified by the colour of Hb, as the degree of molecular SO_2_Hb relates to a certain colour. rHb was quantified using light absorption by the conversion of white light into red light, which is proportional to the concentration of Hb^(^ ^22^ ^)^. Blood flow and rHb are expressed in arbitrary units, whereas SO_2_Hb is expressed as a percentage of O_2_ on Hb. This technology measures the microcirculation of blood from the skin surface to a 2 mm depth.

### Blood chemistry panel

Standard blood chemistry was determined for eligibility testing after an overnight fast using EDTA plasma from the peripheral venous blood using a routine clinical chemistry analyser (Abbott Diagnostics).

### Statistical analyses and sample size calculation

Per-protocol analyses were performed using IBM SPSS for Windows software, version 19.0 (SPSS Inc.). Data are presented as means and standard deviations. Data for pre–post-comparisons were adjusted for plasma volume changes as described elsewhere (except for CP, as it is already expressed in relation to protein concentration)^(^ ^23^ ^)^. Statistical significance was set at *P*< 0·05. The Shapiro–Wilk test was used to determine a normal distribution. Baseline characteristics, performance data, nutrient and clinical chemistry data were compared using the unpaired Student's *t* test. Data obtained for CP, ox-LDL, TOS, MDA, TNF-α, IL-6, SO_2_Hb, rHb and blood flow were analysed using a univariate, three-factorial, repeated-measures ANOVA. Factors were as follows: treatment (FBV or placebo); exercise (pre- and post-exercise); session (walking test 1 and walking test 2). Significant interactions and main effects were analysed by Bonferroni correction.

The sample size estimate of seventeen subjects per group was based on previous data on oxidation and inflammation markers (markers of primary outcome) and subjected to a probability of error (α = 5 %) and to a test power (1 − β = 80 %). Concerning the mean values, we assumed to discover a difference of 20 % between the FBV and placebo groups after 8 weeks of treatment (and in comparison from pre- to post-exercise) and a standard deviation of 20 % for the oxidation markers CP and MDA. For the mean values of TNF-α and IL-6, we assumed to discover a difference of 30 % between the FBV and placebo groups after 8 weeks of treatment (and in comparison from pre- to post-exercise) and a standard deviation of 30 %. Allowing for an anticipated attrition of 20 % in each group, twenty-two subjects per group were recruited to discover the assumed differences.

## Results

### Study population and nutrition

**Fig. 1:**
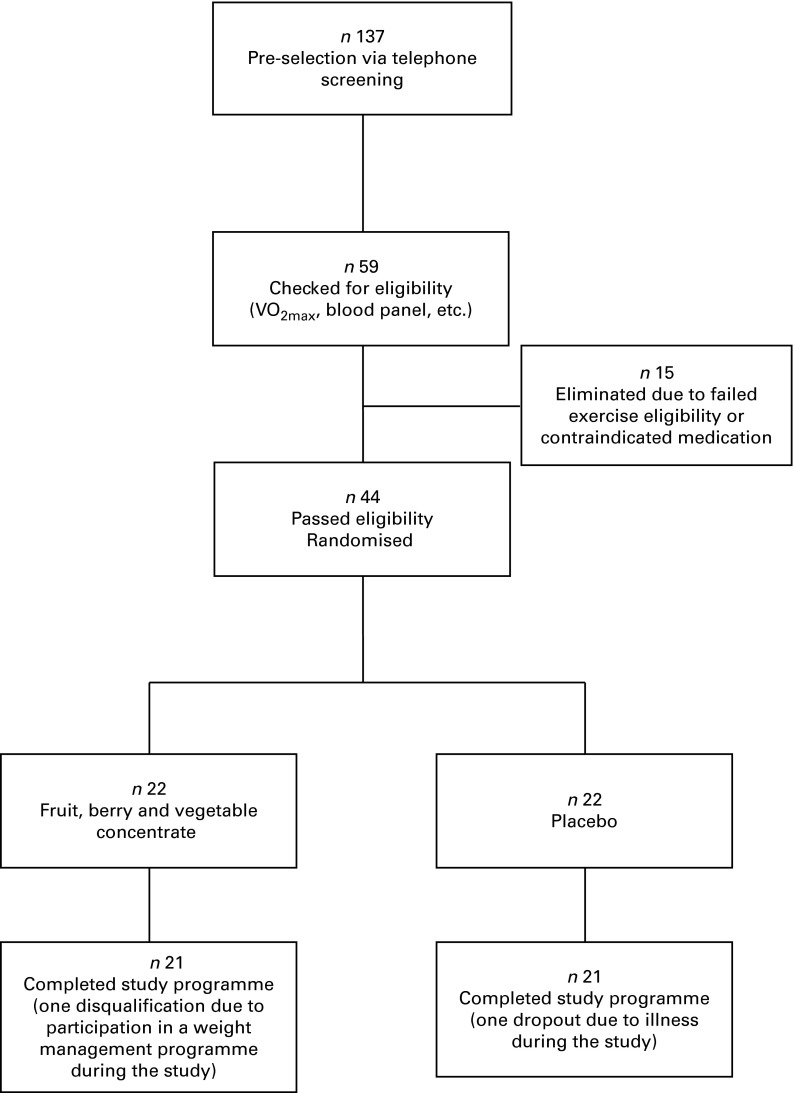
CONSORT (consolidated standards of reporting trials) diagram.

A CONSORT (consolidated standards of reporting trials) diagram outlining participant recruitment is depicted in Fig. 1. Of the forty-four randomised women, forty-two completed the full programme and were included in the statistical analyses. There was one early termination in each study group: in the FBV group, one subject was disqualified at the follow-up visit due to weight loss >3 % of baseline body weight; in the placebo group, one person withdrew due to illness unrelated to the study.

The returned capsule count at the end of the study estimated a compliance >85 % in both groups. The groups did not differ in age, BMI, VO_2max_, VO_2max_ related to body weight, maximum workload (*P*_max_), clinical blood chemistry variables and habitual diet (*P*>0·05; Table 1).

**Table 1:** Baseline characteristics, performance data, clinical chemistry and nutrition data of the forty-two pre-menopausal, obese, but otherwise healthy, women (Mean values and standard deviations)

		FBV (*n* 21)	Placebo (*n* 21)
Variables	Reference values* †	Mean	sd	Mean	sd
Age (years)		40·8	3·7	41·3	4·2
BMI (kg/m^2^)		34·3	4·2	34·6	3·5
Weight (kg)		92·2	12·9	91·6	11·3
VO_2max_ (ml)		2198	127	2259	169
VO_2max_ (ml/kg per min)		25·8	3·5	27·1	3·6
*P*_max_ (km/h)+slope (%)		6+7	1·87	6+7	1·69
*P*_68 %VO_2max__ (km/h)		6	0·62	6	0·55
Clinical chemistry					
Glucose (mmol/l)	3·9–6·1	5·1	1·5	5·0	1·4
Hb (g/l)	115–155	126	13	130	16
Fe (μmol/l)	11–29	16·4	5·5	17·6	5·9
Ferritin (μg/l)	18–300	78·4	55·5	89·4	59·1
Cholesterol (mmol/l)	< 6·35	5·47	1·73	5·56	1·23
HDL (mmol/l)	0·80–2·35	1·27	0·28	1·11	0·43
TAG (mmol/l)	< 1·80	1·27	0·62	1·41	0·56
Average diet/d from the 7 d diet record					
Energy (kJ/d)	7927	7745	454	7923	392
Fat	< 30 % of kJ/d	38·5 %	7·2	38·9 %	8·1
Protein	10–15 % of kJ/d	15·7 %	2·1	16·3 %	3·2
Carbohydrates	>50 % of kJ/d	43·9 %	9·1	43·2 %	10·3
Alcohol (%)	< 3·5	1·9	1·2	1·5	0·9
Water (ml)	2600	2162	95	2022	95
Fibre (g)	30	21	7	19	6
β-Carotene (mg)	4·8	0·14	0·09	0·17	0·11
Fruit and vegetable portions/d	5	1–2		1–2	

FBV, fruit, berry and vegetable juice powder concentrate; *P*_max_, maximal performance; *P*_68 %VO2max_, performance 68 % of maximal oxygen uptake.

* Reference intervals and upper limits for clinical chemistry parameters^(^ ^54^ ^)^.

† Reference values for dietary intake (RDA) in Germany, Austria and Switzerland^(^ ^55^ ^)^.

### 30 min controlled exercise bout

The post-exercise analyses revealed that these women performed at 68·2 (sd 3·1) % of individual VO_2max_. The average walking performance was approximately 6 km/h (Table 1). There were no significant differences between the FBV and placebo groups for these parameters (*P*>0·05).

### Carbonyl proteins

**Fig. 2:**
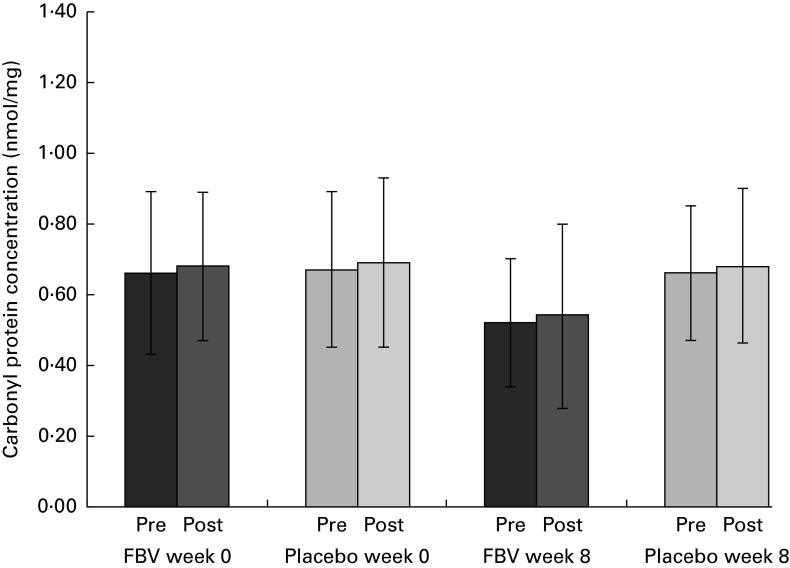
Plasma concentrations of carbonyl groups bound on protein in overweight and obese women (*n* 42) before and after 8 weeks of supplementation, and pre- and post-30 min of walking exercise. Values are means (*n* 21 per group), with standard deviations represented by vertical bars. There was a significant effect of treatment after 8 weeks, with no influence of exercise (*P*= 0·022; ANOVA). FBV, fruit, berry and vegetable concentrate group.

The mean values of both groups were comparable with healthy people of this age (reference interval 0·37–1·16 nmol/mg). There were no differences between the groups at baseline, pre- and post-exercise. After 8 weeks of supplementation, there was a significant difference between the FBV and placebo groups (*P*_Tx_= 0·022; Fig. 2), both pre- and post-exercise. The FBV group had significantly lower CP concentrations compared with the placebo group. The model of exercise had no influence on CP concentrations.

### Oxidised LDL

**Fig. 3:**
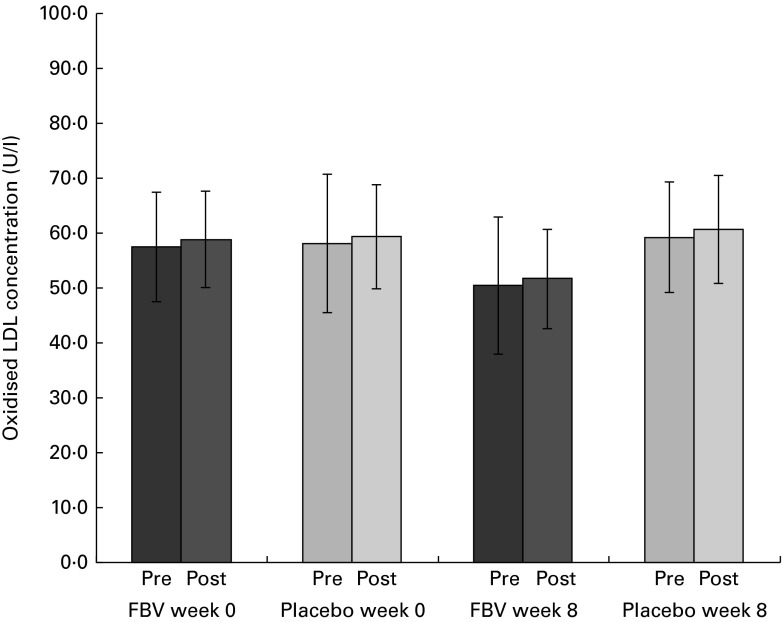
Plasma oxidised LDL concentrations in overweight and obese women (*n* 42) before and after 8 weeks of supplementation, and pre- and post-30 min of walking exercise. Values are means (*n* 21 per group), with standard deviations represented by vertical bars. There was a significant effect of treatment after 8 weeks, with no influence of exercise (*P*= 0·015; ANOVA). FBV, fruit, berry and vegetable concentrate group.

There were no differences between the groups at baseline, but a significant difference after 8 weeks of FBV or placebo supplementation (*P*_Tx_= 0·015; Fig. 3). The FBV group showed lower concentrations compared with the placebo group. The model of exercise had no influence on ox-LDL concentrations. However, all concentrations were within the reference interval, at the beginning and end of the study, pre- and post-exercise (30–80 U/l).

### Total oxidation status

**Fig. 4:**
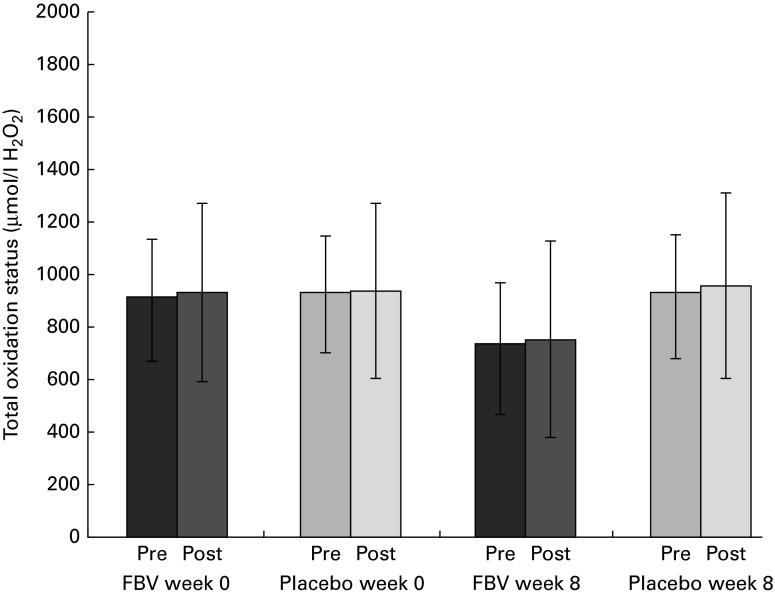
Plasma total oxidation status of lipids in overweight and obese women (*n* 42) before and after 8 weeks of supplementation, and pre- and post-30 min of walking exercise. Values are means (*n* 21 per group), with standard deviations represented by vertical bars. There was a significant effect of treatment after 8 weeks, with no influence of exercise (*P*= 0·010; ANOVA). FBV, fruit, berry and vegetable concentrate group.

There were no differences between the groups at baseline, pre- and post-exercise. After 8 weeks of supplementation, there was a significant difference between the groups (*P*_Tx_= 0·010; Fig. 4). After the supplementation period, FBV supplementation had reduced the elevated baseline values (reference cut-off < 350 μm-H_2_O_2_) from >900 μm-H_2_O_2_ down to approximately 750 μm-H_2_O_2_, which is still above the reference interval. The model of exercise had no influence on TOS.

### Malondialdehyde

There were no differences between the groups at baseline and after 8 weeks of supplementation, pre- and post-exercise, with all concentrations within the reference interval (2·16 (sd 0·29) nmol/ml; data not shown). Also, the model of exercise had no influence on MDA concentrations.

### TNF-α

**Fig. 5:**
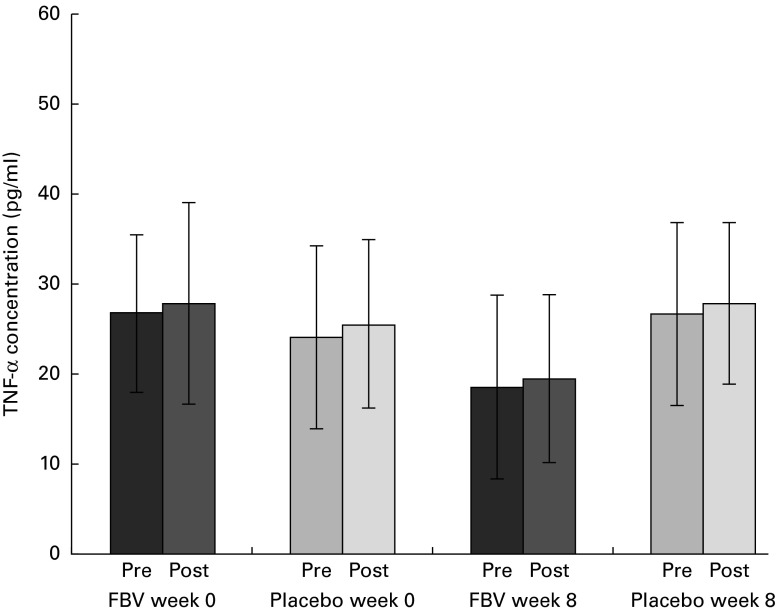
Plasma TNF-α concentrations in overweight and obese women (*n* 42) before and after 8 weeks of treatment, and pre- and post-30 min of walking exercise. Values are means (*n* 21 per group), with standard deviations represented by vertical bars. There was a significant effect of treatment after 8 weeks, with no influence of exercise (*P*= 0·011; ANOVA). FBV, fruit, berry and vegetable concentrate group.

Despite the typically high standard deviation for TNF-α, due to the established cytokine inter-individual variability, the data were normally distributed. There were no differences between the groups at baseline, although pre- and post-exercise concentrations at baseline (mean value >24 pg/ml) exceeded the upper reference limit ( < 20 pg/ml; Fig. 5). Following 8 weeks of FBV supplementation, pre- and post-exercise, TNF-α concentrations were within the normal physiological range, whereas the values remained elevated in the placebo group. Hence, there was a significant difference between the FBV and placebo groups after 8 weeks of intervention. The model of exercise had no influence on TNF-α.

### IL-6

There were no observed differences between the groups at baseline and after 8 weeks of capsule supplementation, both pre- and post-exercise. Also, the model of exercise had no influence. IL-6 concentrations for all subjects remained below the reference cut-off value ( < 11·3 pg/ml; data not shown) throughout the investigation.

### Skin microcirculation

All values were within the normal limits provided by the equipment manufacturer for healthy people. There were no significant differences between the groups at baseline with pre- and post-exercise blood flow. After the 8-week supplement period, blood flow was significantly higher in the FBV group compared with placebo (*P*_Tx_= 0·029). There was also a significant increase due to exercise from pre- to post-exercise (*P*= 0·004) in both groups, at baseline and after 8 weeks.

There were no differences in SO_2_Hb between the groups at baseline, pre- and post-exercise. Following the 8-week capsule supplement period, SO_2_Hb was significantly higher in the FBV group compared with placebo (*P*_Tx_= 0·032), pre- and post-exercise (Table 2). The model of exercise also affected the SO_2_Hb values, with increased values from pre- to post-exercise in both groups at both time points, but this effect did not reach significance (*P*_Ex_= 0·075).

**Table 2:** Microcirculation data from the skin, measured on the back of the hand, between the first and second metacarpal bone* (Mean values and standard deviations, *n* 21 each group)

			FBV week 0 (pre-Ex)	Placebo week 0 (pre-Ex)	FBV week 0 (post-Ex)	Placebo week 0 (post-Ex)	FBV week 8 (pre-Ex)	Placebo week 8 (pre-Ex)	FBV week 8 (post-Ex)	Placebo week 8 (post-Ex)	
Variables	*P*_Tx_	*P*_Ex_	Mean	sd	Mean	sd	Mean	sd	Mean	sd	Mean	sd	Mean	sd	Mean	sd	Mean	sd	Reference value (at rest)
SO_2_Hb (%O_2_Hb)	0·032	0·075	47·1	10·7	47·7	13·9	51·7	8·0	50·3	10·1	54·1†	9·3	48·9	13·4	57·8†	9·7	49·9	12·7	>20
rHb (a.u.)	0·041	0·021	47·2	6·7	47·9	6·6	53·0‡	7·2	55·2‡	7·0	53·6†	6·1	45·5	5·7	59·2† ‡	7·6	53·4‡	5·5	>20
Blood flow (a.u.)	0·029	0·004	14·9	7·5	17·0	9·4	25·2‡	5·8	23·8‡	11·0	20·2†	8·5	15·6	6·3	33·4† ‡	6·6	24·1‡	13·5	>3

FBV, fruit, berry and vegetable concentrate; Ex, exercise; Tx, treatment (FBV or placebo); SO_2_Hb, O_2_ saturation of Hb; O_2_Hb, percentage of O_2_ on Hb; rHb, concentration of Hb; a.u., arbitrary units.

* Mean values are significantly different (*P*< 0·05, ANOVA).

† There was a significant effect of treatment (*P*< 0·05; ANOVA).

‡ There was a significant effect of exercise (*P*< 0·05; ANOVA).

As with SO_2_Hb, there were no differences between the groups at baseline with pre- and post-exercise for rHb. After the 8-week supplement period, rHb was significantly higher in the FBV group compared with placebo (*P*_Tx_= 0·041), pre- and post-exercise (Table 2). There was also a significant effect of exercise to increase rHb from pre- to post-exercise (*P*_Ex =_0·021) in both groups, at baseline and after 8 weeks of treatment.

## Discussion

Increased protein and lipid oxidation, as well as low-grade inflammation, are conditions associated with increased cardiovascular and chronic disease risk^(^ ^1^ ^,^ ^2^ ^,^ ^5^ ^)^. Obese people are at a higher risk of developing chronic medical conditions, thus making an interesting study population. The present investigation was primarily focused on the responses of oxidation, inflammation and capillary microcirculation markers in obese pre-menopausal women, after an 8-week intervention with FBV or placebo capsules, followed by a single bout of aerobic exercise. The resulting data show, after the 8-week study period, (1) compared with placebo, the FBV group had a reduction in the markers of protein oxidation, ox-LDL and total lipid oxidation, and lower concentrations of the chronic inflammation marker TNF-α. The study also revealed that (2) FBV supplementation increased the microcirculation in the skin. (3) A 30 min walking exercise at 70 % of VO_2max_ increased blood flow and rHb in the skin in both groups (secondary outcome) and (4) the walking exercise did not generate additional oxidative stress in these sedentary obese women (tertiary outcome).

### Oxidative stress markers

In the present study, protein oxidation (as assessed by CP) was decreased after the 8-week FBV supplementation period. The decrease, or attenuation, of CP in healthy and trained subjects in response to this FBV supplementation has been reported previously^(^ ^10^ ^,^ ^24^ ^–^ ^26^ ^)^, and this effect is also now demonstrated in these obese women. The bioavailability of FBV antioxidant vitamins and phytonutrients has been reported previously^(^ ^11^ ^,^ ^27^ ^,^ ^28^ ^)^ and may be the explanation for the consistent observation of decreased or attenuated protein oxidation. However, all CP values remained within the reference interval throughout the investigation in all participants.

Similar to CP, ox-LDL was reduced after 8 weeks of FBV supplementation. Ox-LDL is one of the few recognised parameters of the European Food Safety Authority to estimate oxidative damage to lipids^(^ ^29^ ^)^. It is also a recognised cardiovascular risk factor associated with obesity^(^ ^30^ ^–^ ^34^ ^)^. Over the study period, the FBV group had a 12 % reduction in ox-LDL concentrations (from 58 to 51 U/l). A similar reduction has been reported in heavy smokers using the same FBV capsules for a 3-month period^(^ ^35^ ^)^. This consistent finding may be due to the antioxidant activity of FBV, protecting LDL from oxidation.

TOS, another marker of lipid peroxidation, was elevated at all measured time points, indicating higher concentrations of total lipid peroxides in this cohort of obese women. This surrogate marker is a comprehensive indicator of lipid peroxidation, and thus not as specific for the oxidation of certain molecules, unlike ox-LDL or MDA. The elevated TOS values observed here might reflect a higher oxidation state of MUFA or oxidation derived from other sources, such as advanced glycation end products. These substances originate from a fat-rich diet^(^ ^36^ ^)^, which was consumed by the women in the present study (Table 1). The antioxidant functions of FBV decreased TOS concentrations; however, the values remained elevated at the end of the present 8-week study. It would be interesting to observe the response of this marker after long-term FBV supplementation.

MDA is a commonly used marker to estimate lipid peroxidation^(^ ^37^ ^–^ ^39^ ^)^. It is an indicator of damage to PUFA^(^ ^39^ ^)^. Protein-bound MDA was assessed in the present investigation. Neither the capsule treatment nor exercise was distinctive enough to effect changes in MDA concentrations, which remained within the reference interval throughout the study.

### Inflammatory markers

Low-grade chronic systemic inflammation has commonly been reported in obese populations, which is accompanied by increased systemic levels of cytokines including TNF-α and IL-6^(^ ^14^ ^,^ ^27^ ^)^.

The changes in TNF-α concentrations in the FBV group were remarkable. At baseline, both study groups had elevated concentrations, exceeding the reference cut-off value (20 pg/ml). After 8 weeks of supplementation, TNF-α in the FBV group was within the reference limit. This is an important finding in the context of the frequently postulated involvement of TNF-α in obesity and imbalanced insulin metabolism^(^ ^40^ ^–^ ^42^ ^)^. It has been postulated that adipose tissue, which produces TNF-α, is the main source of circulating TNF-α^(^ ^43^ ^,^ ^44^ ^)^. However, it has also been observed that a low intake of β-carotene, found in fruit and vegetables, is inversely related to TNF-α, due to a diet-dependent decreased antioxidant and anti-inflammatory capacity^(^ ^9^ ^)^. In the present study, overweight and obese women had increased TNF-α concentrations at baseline, along with suboptimal dietary intakes of β-carotene and fruit and vegetables (Table 1). We are aware that the lack of serum β-carotene measurements in the present study is a limitation. On the other hand, although β-carotene was not measured, others have consistently reported increased β-carotene concentrations in studies using the same FBV supplementation^(^ ^27^ ^,^ ^28^ ^,^ ^45^ ^)^. Therefore, it is reasonable to expect the FBV group in the present study also had increased β-carotene concentrations, which may have contributed to the decrease observed in TNF-α in the FBV group, particularly since body weight and exercise habits remained constant during the study.

All subjects had IL-6 values within the normal limit and no changes were observed during the study. Other studies have also reported that FBV supplementation did not influence IL-6 concentrations^(^ ^10^ ^,^ ^11^ ^)^, and obviously, the model of exercise was not exhaustive enough to generate IL-6 from muscle inflammation.

### Microcirculation markers

The maintenance of microvascular integrity is related to intact endothelial NO metabolism and protective against adiposity-linked CVD^(^ ^46^ ^,^ ^47^ ^)^. As NO metabolism is also dependent on redox biochemistry, we hypothesised that FBV supplementation might exert the effects on the microcirculation assessed on the easily accessible skin surface.

All skin microcirculation values were within the reference limit at all the time points assessed; however, these values did increase with both FBV supplementation and exercise. The observed increases in blood flow, SO_2_Hb and rHB indicate reduced O_2_ extraction and vasodilation of the blood vessels. In theory, perhaps the constituents of FBV would have stimulated NO metabolism, for better oxygenation in the capillaries near the skin surface. Plotnick *et al.* ^(^ ^48^ ^)^ demonstrated that this FBV provides dietary nitrate, resulting in an increase in nitrate/nitrite levels after a 4-week study of FBV supplementation in healthy volunteers, accompanied by improved flow-mediated dilatation after a high-fat meal. Once NO is generated via nitrate and nitrite reductase, it reduces O_2_ extraction from Hb and also the O_2_ cost in the tissue^(^ ^49^ ^)^. In addition to the nutrients from FBV, it is presumable that exercise affected NO generation via increased blood flow, exerting shear stress on endothelial cell membranes. It has been well established that an increase in blood flow stimulates vascular endothelial cells and promotes the production of various vasodilator substances including NO or prostacyclin^(^ ^50^ ^–^ ^52^ ^)^.

### Effect of exercise on oxidation

A null effect was observed in these women with regard to exercise-generated oxidation. The 30 min exercise bout at close to 70 % of VO_2max_ did not result in increased protein and lipid oxidation (indicated here via CP, ox-LDL, TOS and MDA). This is in line with the recent findings that demonstrated a lack in the increase of CP concentrations with 70 % VO_2max_ exercise for 40 min in trained men^(^ ^53^ ^)^. However, to our knowledge, the present investigation demonstrated for the first time that obese, but otherwise healthy, women can perform walking exercise at 70 % VO_2max_ over 30 min without generating oxidative stress. This suggests that exercise regimens for basically healthy obese women should also include aerobic exercise of higher intensity than is usually applied to accelerate improvements in energy consumption capacity and aerobic fitness.

### Conclusions

The overall result from the present study is that 8 weeks of supplementation with an encapsulated fruit, berry and vegetable concentrate decreased the oxidation of proteins and lipids in plasma and reduced low-grade inflammation in overweight and obese women compared with the placebo group. Further, FBV supplementation and 30 min of aerobic walking exercise complemented each other to promote the microcirculation in the skin. In addition, we demonstrated that this population does not suffer oxidative damage or inflammation after 70 % VO_2max_ walking intensity over 30 min.
